# Analysis of Organophosphorus-Based Nerve Agent Degradation Products by Gas Chromatography-Mass Spectrometry (GC-MS): Current Derivatization Reactions in the Analytical Chemist’s Toolbox

**DOI:** 10.3390/molecules26154631

**Published:** 2021-07-30

**Authors:** Carlos A. Valdez, Roald N. Leif

**Affiliations:** 1 Physical and Life Sciences Directorate, Lawrence Livermore National Laboratory, Livermore, CA 94550, USA; leif1@llnl.gov; 2 Nuclear and Chemical Sciences Division, Lawrence Livermore National Laboratory, Livermore, CA 94550, USA; 3 Forensic Science Center, Lawrence Livermore National Laboratory, Livermore, CA 94550, USA

**Keywords:** nerve agents, GC-MS, derivatization, silylation, methylation, chemical warfare agents, sarin, Novichoks

## Abstract

The field of gas chromatography-mass spectrometry (GC-MS) in the analysis of chemical warfare agents (CWAs), specifically those involving the organophosphorus-based nerve agents (OPNAs), is a continually evolving and dynamic area of research. The ever-present interest in this field within analytical chemistry is driven by the constant threat posed by these lethal CWAs, highlighted by their use during the Tokyo subway attack in 1995, their deliberate use on civilians in Syria in 2013, and their use in the poisoning of Sergei and Yulia Skripal in Great Britain in 2018 and Alexei Navalny in 2020. These events coupled with their potential for mass destruction only serve to stress the importance of developing methods for their rapid and unambiguous detection. Although the direct detection of OPNAs is possible by GC-MS, in most instances, the analytical chemist must rely on the detection of the products arising from their degradation. To this end, derivatization reactions mainly in the form of silylations and alkylations employing a vast array of reagents have played a pivotal role in the efficient detection of these products that can be used retrospectively to identify the original OPNA.

## 1. Introduction

Gas Chromatography-Mass Spectrometry (GC-MS) has been a central analytical technique in the field of Chemical Warfare Agent (CWA) detection, analysis, and identification. The role that this form of mass spectrometric method has provided to analysts immersed in this specific area is invaluable and continues to grow in importance as the CWA molecular space rapidly expands. CWAs represent some of the most toxic chemical entities ever produced in a laboratory. Their sole purpose is to eliminate a threat or incapacitate it [1]. Among the most notorious members in this class of toxic compounds are the organophosphorus-based nerve agents [2]. The lethal effects of nerve agents stem from their efficient inhibition of the enzyme acetylcholinesterase (AChE), which is an enzyme involved in maintaining homeostasis in the nervous system and controls muscle function [3,4]. Inhibition of AChE leads to the accumulation of the neurotransmitter acetylcholine in synaptic junctions that results in complete muscle relaxation, an event that, when affecting those involved in respiration, leads to the demise of the affected individual by asphyxiation.

**Figure 1:**
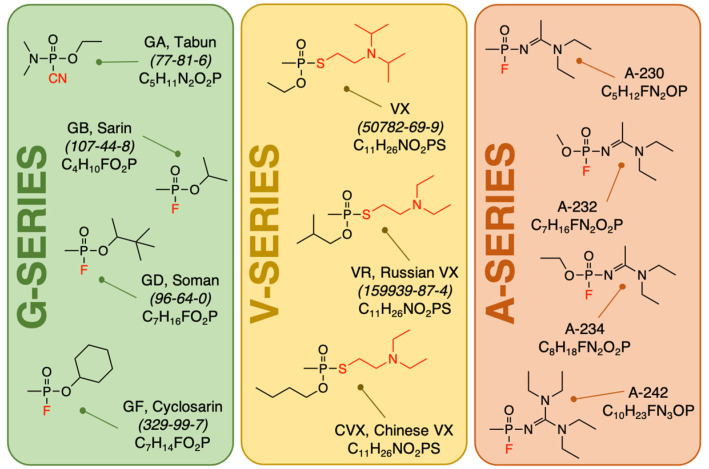
Chemical structures of OPNAs. Degradation products arising from all these three kinds of nerve agents are similar and overlapping in structure.

Traditional OPNAs fall into two main categories since their introduction in the 20th century, being used against military as well as civilian targets and these are the G-series and the V-series (Figure 1) [5,6,7]. The G-series were developed in Germany and hence the G-prefix in their notation, and they share a common chemical core, a pentavalent phosphorus center containing a methyl group, a fluorine atom and an alkyl ester group that ultimately distinguishes each member in this class from one another. Thus, Sarin (or GB) possesses the isopropoxy moiety, while Soman (or GD) possesses the chiral, 2,2-dimethyl-2-butoxy moiety and lastly Cyclosarin (or GF) which contains the cyclohexyloxy moiety. The OPNA Tabun (or GA) is considered a G-series agent even though it does not share many common structural features with the other members in the group. GA possesses a cyanide moiety instead of the fluoride which serves as the leaving group in addition to a dimethylamino moiety instead of a methyl group. The second category, the V-series also consists of a pentavalent phosphorus atom featuring an alkoxy and a methyl substituent in addition to a *N,N*-dialkylaminoethylthiolate group that serves as a leaving group in its reaction with AChE. One of the most famous members in this class is VX (*O*-ethyl-*S*-[2-(Diisopropylamino)ethyl] methylphosphonothioate) that consists aside of a methyl and ethoxy substituents, the hallmark *N,N*-diisopropylaminoethylthiol substituent. Additionally, the other two members in this class, VR (Russian VX) and CVX (Chinese VX) are structural isomers of VX and of each other. In the case of VR, the alkoxy group is the isobutoxy group while the thioalkyl side chain is the *N*,*N*-diethylaminoethylthiol substituent. For CVX, the alkoxy group is the *n*-butoxy group while the thioalkyl side chain is the *N*,*N*-diethylaminoethylthiol substituent. A third category that broke into the world’s spotlight are the A-series or Novichok agents [8] with their use in the poisoning of Sergei Skripal and his daughter in Salisbury, UK in 2018 [9] and the poisoning of Alexei Navalny, who became ill during a flight from Tomsk to Moscow, Russia in 2020 [10]. The structures of these “newcomers” as their name means are very different from the G- and the V-series and also differ significantly among themselves to the point that they do not share the same structural isomerism the V-series do. Although the structural make-up among these three kinds of OPNAs is different, one common factor that unites them all is the presence of a highly reactive, pentavalent phosphorus center. For the Novichok agents, the role of the leaving group is played by the fluorine atom just as in the case of Sarin or Soman. One last reason for the basis of OPNA’s worldwide concern is the fact that established synthetic routes exist for their large-scale production. Fortunately, a great deal of information is known about these routes and the ever-evolving field of chemical forensics is rapidly developing methods involving chemical attribution signature analysis to help forensic analytical chemist determine how a specific OPNA synthesized [11,12,13].

## 2. Degradation Pathways for Nerve Agents

**Figure 2:**
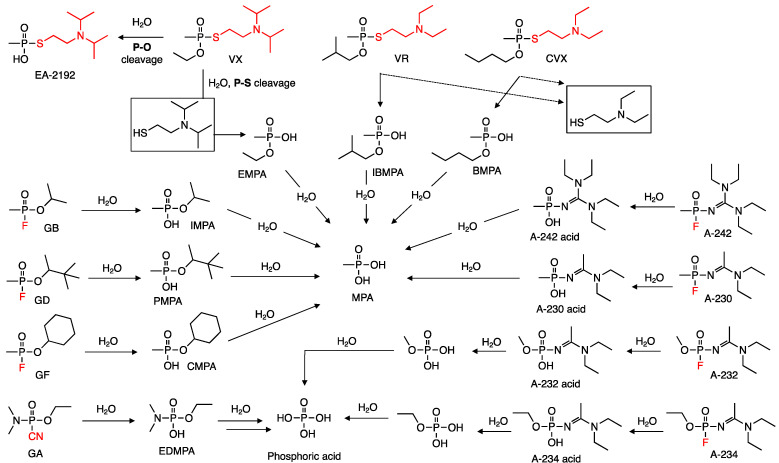
Hydrolytic degradation pathways for OPNAs and intermediary species in the processes. The more reactive leaving group in each agent is denoted in red.

As the threat that nerve agents pose is well recognized, several research efforts from various groups in government and private sectors, have focused on the development of more powerful antidotes [14,15,16,17,18,19], more efficient protective gear for the warfighter [20,21], and more effective methods for their destruction [22]. Due to their different structural features among the G-, V- and A-series, nerve agents do not hydrolyze equally and in some cases one specific set of conditions may lead to the formation of equally toxic by-products. One of the most common, practical, and effective methods for the destruction of nerve agents is the use of bleach (hypochlorite). This oxidative method accomplishes the destruction of most agents in a matter of minutes at ambient temperature [22]. Figure 2 below shows some of the most common pathways for the degradation of the three classes of nerve agents discussed in this review and these involve the normal hydrolysis of these chemicals (i.e., no oxidation). Thus, for the G-series, initial reaction of water with these series leads to the formation of the phosphonic acid monoester via P-F bond scission. A second round of hydrolysis, occurring at a much a faster rate than the first one, involves the hydrolysis of the P-O bond to yield ultimately methylphosphonic acid (MPA). A similar stepwise process can be invoked for the V-series agents, with the first hydrolytic step involving the P-S bond to again yield a phosphonic acid monoester that the undergoes another hydrolytic step to yield MPA. Now, although no published studies involving the hydrolytic pathways for the Novichok agents exists, one can predict that like the G-series, and following the nature of the generated conjugate bases, these agents will undergo scission at the P-F bond initially to generate an amidine/guanidine methylphosphonic acid intermediate that can undergo another round of hydrolysis to ultimately yield MPA.

OPNAs possess of range of values when it comes to their persistence in the environment, whether it involves their residence in various soil matrices or their stance in aqueous solutions including those of biological origin. Throughout the years and after the realization of their potential use in mass destruction, several methods have made their appearance into the light showcasing their abilities to efficiently destroy these toxic substances. Two of the original, main approaches that have found numerous uses are their base-mediated and oxidative degradation. As the Novichok agents are relatively new in the open literature, their environmental or biological degradation pathways are unknown, however the degradation pathway sequence outlined in Figure 2 for these OPNAs is very likely to govern the way they will degrade. As a caveat to these degradation methods, one must consider the fact that a method efficient for a class of NA may not necessarily mean that it will be equally efficient on another kind. During the base-mediated degradation protocols, the use of highly basic aqueous solutions (pH > 11) is employed to degrade the agent, a method that relies on the nucleophilicity of the ^−^OH ion and its attack on the phosphorus center of these chemicals [23]. As expected, G-series agents degrade very rapidly under these conditions to yield the alkylmethylphosphonic acids that thrive under the basic conditions and slowly and eventually degrade to methylphosphonic acid (MPA). With regard to the V-series, the same rapid outcome is observed in highly basic solutions but the regioselectivity for the hydrolysis is not uniformly and solely on the P-S linkage but can also affect the P-O linkage (Figure 2). For example, VX undergoes basic hydrolysis to yield the non-toxic product ethylmethylphosphonic acid (EMPA) and EA-2192 which is equally toxic as VX [24,25,26]. The second method involves the use of bleach as an oxidative source for their degradation and it is the go-to method for their destruction in environmental and laboratory settings. A drawback of the two methods described above is their corrosive nature that needs to be considered when performing the decontamination of very expensive, critical, and specialized army equipment within the military setting. To this end, alternative methods that can accomplish the destruction of OPNAs while minimizing their hydrolytic and oxidative reaction towards the environment have been foci of intense research efforts. Some of these methods include the use of metal-based catalysts that can operate in catalytic fashion at low basic pH ranges [27,28,29,30,31,32,33,34], hydrogen peroxide-based solutions that are not as corrosive as bleach [35,36,37], and metal oxide-based approaches (e.g., FeO, Fe_2_O_3_, Al_2_O_3_, MgO) [38,39,40,41] that also degrade the OPNA via oxidative degradation pathways. Another area of research that has yielded unique methods for the degradation of OPNAs is in the materials sciences, efforts that have yielded technologies such as metal organic frameworks (MOFs) [20,42,43] and the aforementioned second skin technologies [20,21] that can find significant application in the field by providing a strong protective layer to the warfighter. 

## 3. GC-MS as an Important Technique in the Analysis of Nerve Agent Degradation Products

OPNAs in their intact form can be analyzed and detected by GC-MS, with their mass spectra easily corroborated by conducting mass spectral searches against databases such as NIST20, from the National Institute of Standards and Technology (NIST) or OCAD, the OPCW Central Analytical Database from the Organisation for the Prohibition of Chemical Weapons (OPCW) [44,45,46]. However, due to the high reactivity of these chemicals in biological as well as environmental matrices, there is a great chance that analytical chemists will encounter the degradation products rather than the agent itself. Now, detection of these degradation products can be highly useful from a forensic standpoint in retrospectively determining the nature of the original OPNA. Unfortunately, analysis of OPNA degradation products come with its own difficulties as some of these, particularly the low molecular weight phosphonic acids, are difficult to detect by GC-MS in their native state. For this reason, derivatization plays an important role in the analysis of OPNA degradation products as it provides modified analytes with better chromatographic characteristics for GC-MS analysis [47,48,49]. In the sections below, we will discuss the main derivatization strategies that have been developed and employed over the years in the analysis of these degradation products.

Interest in the field of derivatizations during the analysis of not only OPNA degradation products but also those arising from chemical warfare agent (CWA) in general is always strengthened yearly during proficiency tests (PTs) administered by the Organisation for the Prohibition of Chemical Weapons (OPCW). OPCW is an organization based in the Hague, Netherlands and their primary mission is to monitor, control and eliminate chemical weapons. To this end, OCPW has assigned various laboratories around the globe to serve as hubs where the analysis for these chemicals can take place in the case an event involving these toxic chemicals arises. As our laboratory in the Forensic Science Center (FSC) at the Lawrence Livermore National Laboratory (LLNL) is part of this worldwide consortium of laboratories, we participate in yearly PTs to ensure certification by OCPW. From an OPCW proficiency test (PT) standpoint, a participating laboratory needs to identify a reportable analyte by a minimum of two analytical techniques with underivatized and derivatized versions of a compound being acceptable reporting criteria [50,51,52,53,54]. Multiple derivatization methods are useful because having more than one derivatized version of a given analyte is very important in providing strong, unequivocal evidence of its presence in a given matrix.

## 4. Silylation Methods

**Figure 3:**
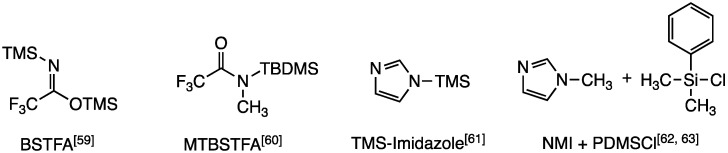
Commonly used silylation reagents and reagent combinations. For MTBSTFA, the reagent is provided with trimethylsilyl chloride (TMCS, 1%) as a catalytic additive. Abbreviations used: TMS = trimethylsilyl-; TBS = *tert*-butyldimethylsilyl-; NMI = N-methylimidazole; PDMSCl = phenyldimethylsilyl-.

Silylation is one of the most widely employed derivatization techniques in the field of GC-MS analysis [55,56,57]. The installation of the silyl group or tag onto polar analytes convert it into derivatives that are suitable for the technique and in some cases these derivatives possess enhanced detection relative to the underivatized analyte. Two of the most common silylating reagents are *N*,*O*-bis(trimethylsilyl)trifluoroacetamide (BSTFA) and *N*-*tert*-butyldimethylsilyl-*N*-methyltrifluoroacetamide (MTBSTFA) but other approaches to install the silyl moiety have found wide applications specially within the scope of OPCW [58,59,60,61,62] (Figure 3). Reaction with BSTFA results in the installation of the trimethylsilyl (TMS) group while reaction with MTBSTFA results in the installation of the *tert*-butyldimethylsilyl group (TBDMS). In terms of stability, the TBDMS group offers derivatives with higher stability than the TMS group towards acid and base hydrolysis [63,64].

A report appearing in 1989 by Purdon et al. from the Department of National Defence in Ottawa, Canada describes their studies on various CWA-related methylphosphonic acid silylations using the *tert*-butyldimethylsilyl group [65]. To this end, the authors compare the silylation efficiency of these acids by three different reagent/reagent combinations namely MTBSTFA, MTBSTFA (with 1% TBDMSCl) and the derivatization kit containing TBDMSCl and imidazole (1:2.5) in DMF. The seven acids included in this study were MPA, ethylphosphonic acid (EPA), *n*-propylphosphonic acid (*n*PPA), *n*-butylphosphonic acid (*n*BPA), EMPA, IMPA and PMPA. The researchers found that all seven acids were quantitatively converted to their respective silyl esters by MTBSTFA and MTBSTFA (with 1% TBDMSCl) at 60 °C and after only 30 min. Conversely, silylation using the derivatization kit (TBDMSCl and imidazole) did not proceed to completion resulting in lower yields of the product available for GC-MS analysis even under more aggressive conditions. The authors found that phosphonic acid quantitation as these derivatives can be carried out down to 500 pg when using the GC coupled to flame-photometric detection (FPD), while when using Electron Impact Gas Chromatography Mass Spectrometry (EI-GC-MS), quantitation values can be lowered to 300–500 pg in full scan mode and even lower, down to 30–60 pg, when using the SIM mode.

In 2007, silylation was also employed for the derivatization of phosphonic acids involved the use of MTBSTFA (with 1% TBDMSCl) to obtain derivatives of various phosphonic acids that were subsequently analyzed by gas chromatography with inductively coupled plasma mass spectrometry (GC-ICPMS) [66]. In this work, Richardson and Caruso describe the analysis of *tert*-butyldimethylsilyl derivatives of seven phosphonic acids that included EMPA (VX acid), IMPA (GB acid), EDPA (GA acid), IBMPA (VR acid), PMPA (GD acid), CMPA (GF acid) and the ultimate end product MPA. ICPMS detection was chosen for this application for its high sensitivity when operated in the ^31^P channel that led to not only to optimal separation and speciation of all TBDMS-modified phosphonic acids within the short timeframe for the GC run (t = 10 min). Further confirmation of each species was accomplished using time-of-flight (TOF) mass spectrometry. The authors found that the most optimal conditions for the derivatization involved the heating of the MTBSTFA (with 1% TBDMSCl) reagent at 80 °C for 45 min. Detection limits determined for this method were found to be <5 pg and successfully applied to the analysis of all seven phosphonic acids when spiked in an aqueous (Miami river) and a soil matrix.

A paper describing an approach combining solid phase extraction followed by solid phase derivatization of various phosphonic acid markers for nerve agents was introduced in 2009 by the Ostin group at the Swedish Defence Research Agency (FOI) [67]. The report described the use of in vial solid phase derivatization (SPD) of nine phosphonic acids using BSTFA (with 1% trimethylsilyl chloride) followed by analysis by GC-MS. The method was found to be very sensitive for the TMS-derivatized products displaying recovery values between 83–101% and a limit of detection (LOD), under the single-ion monitoring (SIM) mode, down to 0.14 ppb. The approach was successfully tested for its ability to unambiguously identify MPA, PMPA, 1-methylpentyl methylphosphonic acid, 4-methylpentyl methylphosphonic acid and IMPA present in aqueous samples administered during the 19th OPCW PT. The phosphonic acids’ concentrations were estimated to range between 5–8 ppm in both aqueous samples showcasing the method’s robustness in correctly identifying these in the sample.

In 2012, a report surfaced from the Swedish Defence Research Agency (FOI) and the Department of Chemistry at Umeå University describing the use of BSTFA (with 1% TMCS) to silylate the toxic degradation products of VX namely S-2-(*N,N*-diisopropylaminoethyl)methylphosphonothiolate (EA-2192) and VR namely S-2-(*N,N*-diethylaminoethyl)methylphosphonothiolate (REA) [68]. VX, and by analogy VR, are known to undergo hydrolysis under basic conditions via two pathways with a major one leading to the formation of non-toxic EMPA and a minor one leading to the formation of the equally toxic EA-2192 [22]. EA-2192 is a particularly difficult analyte to derivatize using derivatization agents [69,70,71] and in this work the authors developed a protocol that involved the initial aqueous extraction of the analyte using a strong anion exchange disk to remove interfering salts. The silylation was carried out after evaporation to dryness and treatment of the residue with BSTFA (with 1% TMCS) in acetonitrile for GC-MS analysis. The LOD values determined for the protocol were 10 ng·mL^−1^ and 100 ng·mL^−1^ for SIMS and full scan mode, respectively. Furthermore, the protocol was employed in the detection of both degradation products in a water sample (W1) from an OPCW PT (19th) and also in spiked water samples (river water and Baltic Bay water).

A recent paper dealing with the derivatization of phosphonic acids using BSTFA was published by Kim et al. from a collaborative effort by the Center of the Cell-Encapsulation Research and the Agency for Defense Development in South Korea [72]. The silylation of the phosphonic acids was done before their efficient extraction using headspace solid-phase microextraction (HS-SPME). The phosphonic acids studied in this work were EMPA, IMPA, CMPA and PMPA and the authors found that the ideal analyte adsorption temperature for their chosen PDMS/DVB SPME fiber was 75 °C. Interestingly, when optimizing the derivatization step, the authors found that increasing the volume of BSTFA resulted in less derivatized product for detection by GC analysis. The method’s limit of detection (LOD) was determined to be between 10–20 pg·mL^−1^ depending on the phosphonic acid under study. Furthermore, the authors go on to show the efficacy of the method in the analysis of PMPA in a polyethyleneglycol-rich matrix sample administered during the 35th OPCW PT. PMPA, present at a 5 μg·mL^−1^ concentration, was detected and unambiguously identified (with the use of a PMPA-TMS standard) in the OPCW sample (sample 351).

## 5. Methylation Methods

Another method of derivatization used in the field of GC-MS, and not limited to the analysis of phosphonic acids, is methylation. As many alkyl groups can be added to an analyte to enhance its detectability by GC-MS, there is one that has found the widest applicability and that is the methyl group [73]. Currently, the reagent used to accomplish the installation of the methyl group on to molecules is diazomethane (DM) [74]. Diazomethane possesses two favorable characteristics that make it a great derivatizing agent, its high reactivity and the fact that its reaction with analytes does not produce any interfering side products. However, its use comes with a concern linked to the explosive hazard associated with its preparation. As DM’s needs to be freshly prepared before its use in sample derivatizations, its explosive hazard becomes a significant concern, although the current methods for its preparation have become fairly safe to conduct [75,76,77]. The associated explosive hazards with DM’s preparation and the need for its constant preparation, have compelled analytical chemists to seek new derivatization agents that can carry out the same transformation (i.e., methylation). To this end, reagents such as trimethylsilyldiazomethane (TMS-DM) [78] and trimethyloxonium tetrafluoroborate (TMO) [79] have found general applicability in GC-MS analyses.

In the field of OPNA degradation product analysis, derivatization in the form of methylation has been widely a useful technique in the GC-MS analysis of these compounds. In 2002, a report by Driskell et al. from the Centers of Disease Control (CDC) in Atlanta described the use of DM for the methylation of five phosphonic acids originating from the degradation of Sarin, Soman, Tabun, Cyclosarin and VX [80]. The authors make use of isotope-dilution GC-MS-MS to analyze all these acids as their methyl esters and report a LOD value of <4 μg·mL^−1^, with the exception of Tabun acid for which the LOD was determined to be <20 μg·mL^−1^ in urine.

In 2004, another report from the CDC described the use of the same isotope-dilution GC-MS-MS approach to analyze the nerve agent metabolites from Sarin, Soman, Cyclosarin, VX and Russian VX in urine [81]. The phosphonic acid hydrolysis products arising from these agents were all derivatized using DM. The authors report that the method’s LOD for all phosphonic acid was <1 μg·L ^−1^, an improvement from their report two years earlier. An interesting methylation approach was introduced in 2011 by scientists at the Organisation for the Prohibition of Chemical Weapons (OCPW) in the Netherlands [82]. In this report, the authors make use of thermally assisted methylation (TAM), using an injector port temperature of 250 °C, followed by silylation using BSTFA to assist in the detection of a panel of phosphonic acids products related to the nerve agents that included EMPA, PMPA, MPA and EPA in addition to benzilic acid which is a marker for the incapacitating agent 3-quinuclidinyl benzilate (BZ) [83,84]. The work compares the performance of TAM using various methylation agents such as trimethylphenylammonium hydroxide (TMPAH) and trimethylsulfonium hydroxide (TMSH). The authors found that methylation using TAM with these reagents provided chromatograms significantly noisier than when using only DM. In addition, the report describes the approach of using TAM in conjunction with silylation (using BSTFA) in order to obtain as much information as possible on a given sample as the use of TMS derivatives is the principal method used for on-site analysis. The method’s LOD for TMPAH was found to be <0.5 ng per injection.

**Figure 4:**
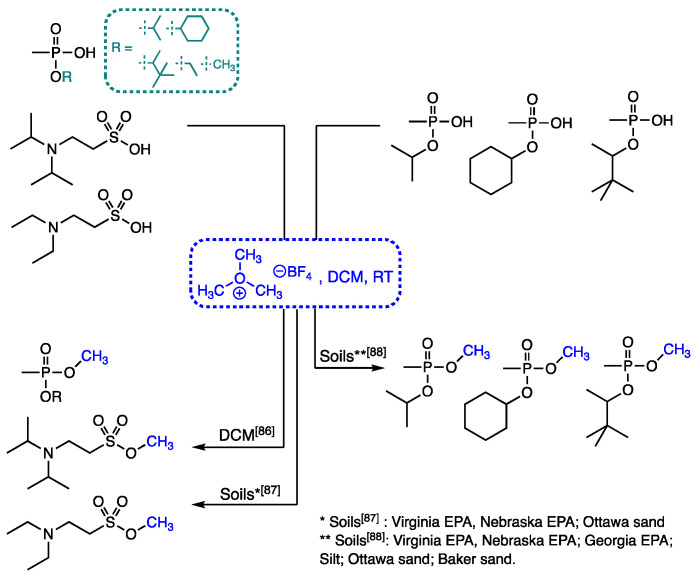
Derivatization approach using trimethyloxonium tetrafluoroborate (TMO) to methylate phosphonic and sulfonic acids related to OPNAs developed in our laboratory.

Since 2016, our group at the Forensic Science Center (FSC) at the Lawrence Livermore National Laboratory (LLNL) has reported the use of the salt TMO in the derivatization of phosphonic acids related to OPNAs [85,86,87] (Figure 4). Our interest in this salt originated from our knowledge that alkyloxonium salts, out of which Meerwein’s reagent (triethyloxonium tetrafluoroborate) is the most famous, have found a plethora of applications in the field of total synthesis due to their reactivity and thus their ability to alkylate hindered hydroxyl [88,89,90] and carboxylic acid groups [91,92,93]. Some key characteristics of the reagent sparked our interest in testing as potential option to DM during analyte methylation reactions for GC-MS analyses. Some of these unique and important characteristics include its salt form, its non-explosive nature, and the generation of minimal interferences in the GC-MS analysis after its use. Thus, in our 2016 report we described the efficient methylation of EMPA, CMPA, and PMPA when present in low concentrations (10 μg·mL^−1^) in DCM. Furthermore, the method was applied to their methylation when these were spiked (at a 10 μg·mL^−1^ concentration each) in a fatty acid ester-rich organic matrix that was featured during the 38th OCPW PT. An interesting observation in this report was the fact that the marked insolubility of the salt in DCM did not have a negative effect on the overall derivatization. In addition to these three representative phosphonic acids, the methylation of the sulfonic acids deriving from the oxidative degradation of VX and VR, *N,N*-diethylamino ethanesulfonic acid (VR-SA) and *N,N*-diisopropylamino ethanesulfonic acid (VX-SA), was also demonstrated.

## 6. Additional, Alternate Derivatization Methods

Other derivatization approaches for the analysis of OPNA degradation products have been introduced throughout the years and continue to be used in the field in parallel to the more established derivatization protocols described above. In 1991, a report from the US Army Medical Research Institute of Chemical Defense (USAMRICD) described the use of pentafluorobenzyl bromide (PFBBr) to derivatize IMPA, PMPA and CMPA [94]. Analysis and detection of the derivatized phosphonic acids was accomplished using electron ionization as well as chemical ionization GC-MS. The three phosphonic acids were detected in urine and blood samples when spiked at a concentration range of 10–200 ng·mL^−1^. Interestingly, detection of the PA products was fairly simple in the urine samples, but proper detection of these in the blood sample was only accomplished after the plasma was isolated and analyzed. 

A report appearing in 1995 by Fredriksson et al. from the National Defence Research Establishment (NDRE) in Sweden described the use of pentafluorobenzyl bromide to derivatize alkyl methylphosphonic acids related to nerve agents [95]. The panel of acids used in the study included EMPA, IMPA and PMPA. The alkylation of the acids spiked in serum, blood, aqueous matrices, and soil was carried out in acetonitrile and after cleaning of the sample by prewashing through a Bond Elut SAX cartridge. Detection and quantification of the acids was done by using gas chromatography negative ion chemical ionization GC/NIMI-MS and GC/NIMI-MS-MS. The authors report detection levels down to the femtogram levels.

In 1995, the Forensic Science Laboratory from the Osaka Prefectural Police Headquarters in Japan published their studies on the derivatization of various phosphate species that included some related to nerve agents using pentafluorobenzyl bromide (PFBBr) in the presence of various polymeric phase transfer catalysts (PTCs) [96]. The phosphonic acid panel included dimethyl phosphate (DMP), diethyl phosphate (DEP), dimethyl thiophosphate (DMTP), diethyl thiophosphate (DETP), dimethyl dithiophosphate (DMDTP) and diethyl dithiophosphate (DEDTP). A total of five PTCs were evaluated and tri-n-butylmethylphosphonium bromide was found to be most efficient catalyst for the pentafluorobenzylation. The actual derivatization takes place in a three-phase reaction mixture where the phosphonic acids present in an aqueous environment are partitioned between a buffer (phosphate, pH 6.5) and toluene (containing the PFBBr) and treated with the polymer-bound PTC. Therefore, as the phosphonic acid derivatives are generated, they get extracted into the organic phase. The authors complete their work by applying the protocol to the derivatization and detection of these acids in river water and urine matrices. 

In 1997, a landmark report describing the direct detection of sarin from blood sample from victims from the Tokyo attack was published by the Netherlands Organisation for Applied Scientific Research (TNO, Prins Maurits Laboratory) [97]. In this work Polhuijs et al. use the fluoride regeneration protocol on the victim’s blood samples to produce sarin that was then identified by high resolution SIM GC-MS and GC-MS coupled to an alkali flame ionization detector. The amount of regenerated sarin (from butyrylcholinesterase, BChE) was <4.1 ng·mL ^−1^ of plasma, a lower value when compared to the baseline detection of this agent in previous studies involving BChE of ~13 ng·mL^−1^. A reason for the low concentrations could be the aging of the samples that were not analyzed by TNO until 10 months after the attack.

In 1999, a report by Miki et al. [98] from the Forensic Science Laboratory at the Osaka Prefectural Police Headquarters in Japan appeared using their tri-*n*-butylmethylphosphonium bromide PTC discovered a few years back in their laboratory [96]. In this work, the alkylmethylphosphonic acids were pentafluorobenzylated using their PTC system, a reaction that occurs as discussed above in a triphasic reaction mixture (liquid-liquid-solid). The three phosphonic acids were EMPA, IMPA and PMPA and analysis as their pentafluorobenzylated esters were accomplished by negative ion chemical ionization (NICI/GC-MS). Notably, the limit of detection reported for this protocol was 60 pg·mL ^−1^.

In 2005, a report from the Defence Science and Technology Laboratory (DSTL) in the United Kingdom described the use of a benchtop ion trap GC-MS for the detection of phosphonic acids related to nerve agents as their pentafluorobenzyl esters in spiked urine samples [99]. The panel of phosphonic acids studied included IMPA, IBMPA, PMPA, CMPA and EMPA and after their extraction (SPE) from the urine matrix, they were alkylated using pentafluorobenzyl bromide. The GCMS was operated in the negative ion mode (NICI/GC-MS) thus allowing for a highly sensitive method for the detection of the derivatized acids. The protocol’s LOD were determined to be 0.1 ng·mL^−1^ for all acids except for EMPA which was found to be 0.5 ng·mL^−1^, this was attributed to the poor recovery of the acid during the method’s extraction section. 

In 2010, a report from FOI explored the use of novel aryldiazomethane-based derivatizing agents to cleverly derivatize phosphonic acids related to OPNAs in a water matrix [100]. The derivatized acids were detected using NICI/GC-MS and further identified by EI-GC-MS. The method’s LOD using NICI using SIM was determined to be ~5–10 ng·mL^−1^ in the aqueous sample while for identification using full scan EI was 100 ng·mL^−1^. The motivation behind the design of these aryldiazomethane agents stems from the idea of producing a reagent that is equally reactive as diazomethane in alkylating the phosphonic acid and in the process installing a fluorinated tag that could be used for enhanced GC-MS detection. In contrast to PFBBr that can be installed in the presence of a base and aided by heating, these aryldiazomethane agents would be so reactive that only mild heating would be needed and analogously to diazomethane would produce very little by-products in the process. Out of the four studied reagents, 1-(diazomethyl)-3,5-bis(trifluoromethyl)benzene was found to be most robust and consistently performed well during their reaction optimization phase and when it was tested for its efficacy in an OPCW PT (19th) aqueous sample (W1) spiked with PMPA, 1-methylpentyl-MPA, 4-methylpentyl-MPA and MPA.

In 2013, Nyholm et al., at FOI disclosed another remarkable report this time involving the use of 3-pyridyldiazomethane to yield picolinyl ester derivatives of a panel of phosphonic acids [101]. Some of the phosphonic acids used in the evaluation of the protocol were direct hydrolysis products of G- and V-series nerve agents and included EMPA (VX), IMPA (GB), BMPA (CVX), IBMPA (VR), PMPA (GD) among others. The reaction was found to be efficient at derivatizing all the phosphonic acids when conducted for 90 min at 60 °C. The detection of the derivatized phosphonic acids was accomplished using EI-GC-MS as well as CI-GC-MS (in both the positive and negative ion modes). After the optimization of the protocol was done, its performance of the protocol was applied to the successful derivatization of MPA (at 5 μg·mL^−1^) and PMPA (at 6 μg·mL^−1^) in a water sample administered during the 19th OCPW PT.

In 2013, another report from FOI described the use of 1-(diazomethyl)-3,5-bis(trifluoromethyl)benzene to derivatize a set of six phosphonic acids that included EMPA, IMPA, PMPA, CMPA and MPA [102]. The derivatives were analyzed by GC–MS and NICI/GC-MS-MS. The selectivity and sensitivity of analyses performed by low- and high-resolution single ion monitoring MS-mode were compared with those performed by multiple reaction monitoring MS-MS-mode. The authors found that the MS-MS technique offered the greatest sensitivity and selectivity, with LODs ranging from 0.5–1 ng·mL^−1^ of urine. Lastly, the method’s robustness was evaluated using urine samples from the 2nd OPCW biomedical confidence building exercise and all phosphonic acids present in the samples were conclusively identified. 

In 2014, a report by Lin et al. from the Academy of Military Sciences in China, demonstrated the established benefit of using pentafluorobenzylation as a means for derivatizing phosphonic acids related to CWAs [103]. In their report, the authors developed and validated a method that detects four phosphonic acids (EMPA, IMPA, IBMPA and PMPA) as their pentafluorobenzyl esters using isotope-dilution GC-MS. The acids were quantified using NICI/GC-MS and found to have a LOD of 0.02 ng·mL ^−1^ when dealing with only a 0.2 mL urine sample volume. Table 1 compiles all the derivatization approaches described above and has been assembled as a quick reference and notes on such work for the reader’s convenience. Careful attention has been given to provide brief description of each GC-MS method employed as well as the nature of the chemical derivatization used in each work.

## 7. Outlook and Concluding Remarks

Within the field of GC-MS derivatization reactions continue to appear that provide invaluable assistance to the analytical chemist in the analysis of phosphonic acids and polar compounds related to OPNAs. Established methods such as silylation employing BSTFA and MTBSTFA and methylation using diazomethane still continue to serve as the preferred ways of modifying polar compounds and transforming them into analytes suitable for GC-MS analysis. However, as advances in instrumentation methods for detection improve, so have the chemical methods that can be used to modify analytes in not only a more efficient manner but in a way to enhance their ease of detection particularly when present at low levels (<1 ppm). The appearance of new derivatizing agents as well as the application of reagents commonly used during total synthesis maneuvers, have become a much welcomed event specifically in instances where the presence of a given analyte in a sample must be corroborated independently by several analytical techniques. With regard to this last point, this path of discovery has had a major impact during the OPCW Proficiency Tests where a low-level analyte related to CWAs needs to be not only identified but its existence in a sample vetted by multiple techniques. It is important for not only laboratories participating in these year-round PTs to continue searching for efficient derivatization reactions but also for more efficient methodologies and sample preparation protocols that can aid in reducing the time needed for the unequivocal identification of these important CWA markers.

## Figures and Tables

**Table 1:** Summary of derivatization methods for phosphonic acids related to OPNAs discussed in this review.

Derivatizing Agent	Analytes	Matrix	GC-MS Method	Method’s Performance	Ref.
MTBSTFA, MTBSTFA (with 1% TBDMSCl) and TBDMSCl:imidazole (1:2.5) in DMF	MPA, ethylphosphonic acid (EPA), *n*-propylphosphonic acid (nPPA), *n*-butylphosphonic (nBPA), EMPA, IMPA and PMPA	Aqueous sample	GC-MS/ICPMS	Quantitation down to 500 pg by GC-FPD. Quantitation for EI-GC-MS (300–500 pg (full scan mode) and 30–60 pg (SIM)).	[65]
MTBSTFA (with 1% TBDMSCl)	EMPA, IMPA, EDPA, IBMPA, PMPA, CMPA and MPA	Aqueous (river water) and soil	GC-MS/ICPMS	GC run total run time = 10 min. LOD < 5 pg	[66]
BSTFA	Nine phosphonic acids that included OPNA-related acids MPA, IMPA and PMPA.	Aqueous (OPCW PT)	GC-MS-SIM	LOD~0.14 ppb (SIM). Detection of IMPA, MPA, PMPA in aqueous OPCW PT sample (19th) present in the range 5–8 μg·mL^−1^.	[67]
BSTFA (with 1% TBDMSCl)	EA-2192, REA	Organic (ACN)	GC-MS-SIM	LOD = 10 ng·mL^−1^ (SIM); 100 ng·mL^−1^ (full scan mode).	[68]
BSTFA	EMPA, IMPA, CMPA and PMPA	Organic (DCM)	EI-GC-MS	LOD = 10–20 pg·mL^−1^. Detection of PMPA in glycerol-rich matrix OPCW PT sample (35th) present at a 5 μg·mL^−1^.	[69]
DM	Sarin, Soman, Tabun, Cyclosarin and VX	Urine	GC-MS-MS (Isotope dilution).	LOD < 4 μg·mL^−1^, with the exception of Tabun acid for which the LOD was determined to be < 20 μg·mL^−1^ in urine.	[80]
DM	IMPA, PMPA, CMPA, EMPA, IBMPA	Urine	GC-MS-MS (Isotope dilution).	LOD < 1 μg·L^−1^.	[81]
DM and TMPAH (TAM) followed by BSTFA	EMPA, PMPA, MPA, EPA and BA	Various organic solvents	EI-GC-MS and GC-FPD	Silylation followed TAM to obtain yet another set of derivatives. LOD (TMAPH) < 0.5 ng.	[82]
TMO·BF_4_	EMPA, CMPA and PMPA	Organic (DCM).	EI-GC-MS	All three acids detected when spiked in fatty acid-rich matrix from OPCW PT (38th).	[85]
TMO·BF_4_	EMPA, CMPA, PMPA, VX-SA and VR-SA	Soils (Ottawa sand, Nebraska, and Virginia Type A).	EI-GC-MS	All five acids detected when spiked in all three soils at a 10 μg·g^−1^ concentration.	[86]
TMO·BF_4_	EMPA, CMPA and PMPA	Soils (Ottawa and Baker sands, Nebraska, Virginia Type A, and Georgia soils and silt).	EI-GC-MS	All three acids detected when spiked in all six soils at a 10 μg·g^−1^ concentration. PMPA detected in soil matrix from OPCW PT (44th) present at a 5 μg·g^−1^ concentration.	[87]
PFBBr	EMPA, IMPA, BMPA, PMPA and MPA	Serum, urine, aqueous and soil.	NICI/GC-MS and NICI/GC-MS-MS	Low femtograms levels detected.	[95]
PFBBr w/PTC	DMP, DEP, DMTP, DETP, DMDTP and DEDTP	River water and urine	GC-MS coupled to Flame Ionization and Electron Capture detection	Five PTCs were evaluated 45 °Cfor thiophosphates 90 °C for non-thiophosphate	[96]
Fluoride Regeneration	Sarin from BChE	Blood from Japan Subway attack victims	SIM-GC-MS and GC-MS	Sarin concentration detected < 4.1 ng·mL^−1^ of plasma	[97]
PFBBr w/PTC	EMPA, IMPA and PMPA	Urine, serum, and saliva	EI-GC-MS andNICI/GC-MS	Detection limits of 50 ng·mL^−1^ (full scan mode), 2.5–10 ng·mL^−1^ (SIM mode), and 60 pg·mL^−1^ for NICI/GC-MS mode.	[98]
PFBBr	IMPA, IBMPA, PMPA, CMPA and EMPA	Urine sample	NICI/GC-MS	SPE on urine sample, followed by GC-MS analysis, LOD = 0.1 ng·mL^−1^ for all acids except for EMPA (0.5 ng·mL^−1^).	[99]
1-(diazomethyl)-3,5-bis(trifluoromethyl)benzene	PMPA, 1-methylpentyl-MPA, 4-methylpentyl-MPA and MPA	Spiked Aqueous OPCW PT (19th) sample.	NICI/GC-MS; EI-GC-MS	LOD = 5–10 ng·mL^−1^) using NICI-SIM; 100 ng·mL^−1using^ (full scan mode.	[100]
3-pyridyldiazomethane	EMPA, IMPA, BMPA, IBMPA, PMPA and others	Aqueous OPCW PT (19th) sample.	EI-GC-MS; CI-GC-MS (^+^/^–^ mode).	MPA (at 5 μg·mL^−1^) and PMPA (at 6 μg·mL^−1^) in OPCW sample.	[101]
1-(diazomethyl)-3,5-bis(trifluoromethyl)benzene	EMPA, IMPA, CMPA, PMPA and MPA	Urine (OPCW Biomedical Sample)	NICI/GC-MS-MS	LOD = 0.5–1 ng·mL^−1^.	[102]
PFBBr	EMPA, IMPA, IBMPA and PMPA	Urine	NICI/GC-MS	LOD = 0.02 ng·mL^−1^ (using 0.2 mL urine)	[103]

## Abbreviations

AChE	acetylcholinesterase
BSTFA	N,O-bis(trimethylsilyl)trifluoroacetamide
BChE	butyrylcholinesterase
BA	benzylic acid
CDC	Centers for Disease Control and Prevention
CMPA	cyclohexyl methylphosphonic acid (GF acid)
CVX	Chinese VX; O-Butyl-S-[2-(diethylamino)ethyl] methylphosphonothioate
CWA	chemical warfare agent
DETP	diethyl phosphate
DM	diazomethane
DMDTP	diethyl dithiophosphate
DSTL	Defence Science and Technology Laboratory (UK)
EA-2192	S-2-(*N,N*-diisopropylaminoethyl)methylphosphonothiolate
EMPA	ethyl methylphosphonic acid (VX acid)
EPA	ethylphosphonic acid
FOI	Swedish Defence Research Agency
FPD	flame photometric detection
FSC	Forensic Science Center
GA	Tabun
GB	Sarin
GD	Soman
GF	Cyclosarin
HS-SPME	headspace solid-phase microextraction
ICPMS	inductively coupled plasma mass spectrometry
IMPA	isopropyl methylphosphonic acid (GB acid)
IBMPA	isobutyl methylphosphonic acid (VR acid)
LNLL	Lawrence Livermore National Laboratory
LOD	limit of detection
MPA	methylphosphonic acid
MTBSTFA	N-tert-butyldimethylsilyl-N-methyltrifluoroacetamide
NDRE	National Defence Research Establishment in Sweden
NICI/GC-MS	negative ion chemical ionization gas chromatography
nBPA	n-butylphosphonic acid
nPPA	n-propylphosphonic acid
OPCW	Organisation for the Prohibition of Chemical Weapons
OPNA	organophosphorus-based nerve agents
PFBBr	pentafluorobenzyl bromide
PMPA	pinacolyl methylphosphonic acid (GD acid)
PT	proficiency test (OPCW)
PTC	phase transfer catalysis
REA	S-2-(*N,N*-diethylaminoethyl)methylphosphonothiolate
SIM	single ion monitoring
TBDMSCl	tert-butyldimethylsilyl chloride
TMCS	trimethylchlorosilane
TMO	trimethyloxonium tetrafluoroborate
TNO	Netherlands Organisation for Applied Scientific Research
TMS-DM	trimethylsilyldiazomethane
USAMRICD	US Army Medical Research Institute of Chemical Defense
VR	Russian VX; O-isobutyl-S-[2-(Diethylamino)ethyl] methylphosphonothioate
VR-SA	*N,N*-diethylamino ethanesulfonic acid
VX	O-ethyl-S-[2-(Diisopropylamino)ethyl] methylphosphonothioate
VX-SA	*N,N*-diisopropylamino ethanesulfonic acid
